# Involvement of NDPK-B in Glucose Metabolism-Mediated Endothelial Damage via Activation of the Hexosamine Biosynthesis Pathway and Suppression of O-GlcNAcase Activity

**DOI:** 10.3390/cells9102324

**Published:** 2020-10-19

**Authors:** Anupriya Chatterjee, Rachana Eshwaran, Gernot Poschet, Santosh Lomada, Mahmoud Halawa, Kerstin Wilhelm, Martina Schmidt, Hans-Peter Hammes, Thomas Wieland, Yuxi Feng

**Affiliations:** 1 Experimental Pharmacology Mannheim, European Center for Angioscience, Medical Faculty Mannheim, Heidelberg University, 68167 Mannheim, Germany; anupriya.chatterjee@medma.uni-heidelberg.de (A.C.); rachana.eshwaran@medma.uni-heidelberg.de (R.E.); santosh.lomada@medma.uni-heidelberg.de (S.L.); halawa@stud.uni-heidelberg.de (M.H.); thomas.wieland@medma.uni-heidelberg.de (T.W.); 2 Centre for Organismal Studies (COS), 69120 Heidelberg, Germany; gernot.poschet@cos.uni-heidelberg.de; 3 Angiogenesis & Metabolism Laboratory, Max Planck Institute for Heart and Lung Research, 61231 Bad Nauheim, Germany; kerstin.wilhelm@mpi-bn.mpg.de; 4 Department of Molecular Pharmacology, University of Groningen, 9713 AV Groningen, The Netherlands; m.schmidt@rug.nl; 5 Groningen Research Institute for Asthma and COPD, GRIAC, University Medical Center Groningen, University of Groningen, 9713 GZ Groningen, The Netherlands; 6 5th Medical Clinic, Medical Faculty Mannheim, Heidelberg University, 68167 Mannheim, Germany; hans-peter.hammes@medma.uni-heidelberg.de; 7 DZHK (German Centre for Cardiovascular Research), partner site Heidelberg/Mannheim, Germany

**Keywords:** nucleoside diphosphate kinase B, Ang-2, O-GlcNAcylation, UDP-GlcNAc, OGA

## Abstract

Our previous studies identified that retinal endothelial damage caused by hyperglycemia or nucleoside diphosphate kinase-B (NDPK-B) deficiency is linked to elevation of angiopoietin-2 (Ang-2) and the activation of the hexosamine biosynthesis pathway (HBP). Herein, we investigated how NDPK-B is involved in the HBP in endothelial cells (ECs). The activities of NDPK-B and O-GlcNAcase (OGA) were measured by in vitro assays. Nucleotide metabolism and O-GlcNAcylated proteins were assessed by UPLC-PDA (Ultra-performance liquid chromatography with Photodiode array detection) and immunoblot, respectively. Re-expression of NDPK-B was achieved with recombinant adenoviruses. Our results show that NDPK-B depletion in ECs elevated UDP-GlcNAc levels and reduced NDPK activity, similar to high glucose (HG) treatment. Moreover, the expression and phosphorylation of glutamine:fructose-6-phosphate amidotransferase (GFAT) were induced, whereas OGA activity was suppressed. Furthermore, overall protein O-GlcNAcylation, along with O-GlcNAcylated Ang-2, was increased in NDPK-B depleted ECs. Pharmacological elevation of protein O-GlcNAcylation using Thiamet G (TMG) or OGA siRNA increased Ang-2 levels. However, the nucleoside triphosphate to diphosphate (NTP/NDP) transphosphorylase and histidine kinase activity of NDPK-B were dispensable for protein O-GlcNAcylation. NDPK-B deficiency hence results in the activation of HBP and the suppression of OGA activity, leading to increased protein O-GlcNAcylation and further upregulation of Ang-2. The data indicate a critical role of NDPK-B in endothelial damage via the modulation of the HBP.

## 1. Introduction

Hyperglycemia increases glucose uptake and metabolism and thus also increases the flux through the hexosamine biosynthesis pathway (HBP), leading to vascular damage. The HBP is an offshoot of glycolysis in which the glycolysis intermediate fructose-6-phosphate is utilized to generate uridine diphosphate *N*-acetylglucosamine (UDP-GlcNAc) in a series of biochemical reactions that involve amino acid, fatty acid, and nucleotide metabolism [1,2,3]. Amongst the enzymes involved in the HBP, glutamine:fructose-6-phosphate amidotransferase (GFAT) is rate-limiting as it catalyzes the initial step of formation of glucosamine-6-phosphate and thus controls UDP-GlcNAc availability [4,5]. Subsequently, UDP-GlcNAc is used as a substrate by O-GlcNAc transferase (OGT) for the reversible attachment of the O-linked *N*-acetylglucosamine (O-GlcNAc) moiety to serine/threonine residues of proteins, a process known as protein O-GlcNAcylation. The counteracting enzyme, O-GlcNAcase (OGA), unselectively catalyzes the removal of O-GlcNAc moieties. Its regulation, however, is still not well understood [6]. Both proteins are well known as the enzymes performing O-GlcNAc cycling in cells. As a reversible posttranslational modification for a wide range of proteins, protein O-GlcNAcylation can alter the activity, interaction, and function of the targeted proteins [7,8,9,10,11,12].

In diabetic retinopathy, one of the major microvascular complications of diabetes, the expression and secretion of endothelial angiopoietin 2 (Ang-2) is elevated, promoting pericyte dropout from the retinal microvasculature and, subsequently, vasoregression [13,14,15]. Hyperglycemia promotes the modification of the transcriptional regulator mSin3A by methylglyoxal in endothelial cells (ECs), triggering the increased O-GlcNAcylation of another transcriptional regulator, Sp3, via recruitment of OGT. Modification of Sp3 diminishes its binding to the Ang-2 promoter, thereby permitting increased Ang-2 expression [12]. Protein O-GlcNAcylation in retinal ECs and pericytes increases in response to high glucose (HG) in a concentration-dependent manner, and the overall O-GlcNAcylation in the retinae of diabetic mice is significantly elevated [16].

Nucleoside diphosphate kinase B (NDPK-B) belongs to the enzyme family of NDPKs, housekeeping enzymes in nucleotide homeostasis, which catalyze the transfer of a γ-phosphate group between a nucleoside triphosphate (NTP), usually ATP, and a nucleoside diphosphate (NDP) via a ping-pong mechanism [17,18]. In the last decade, it has become evident that NDPK-B is essential for the maintenance of normal vascular function [19]. NDPK-B depletion impaired vascular endothelial growth factor receptor 2 (VEGFR-2) and VE-cadherin signaling in ECs [20]. Additionally, NDPK-B^−/−^ mice developed retinal vasoregression similar to diabetic retinae, but without hyperglycemia. Both processes are initiated by the upregulation and secretion of Ang-2 [21]. Similar to HG treatment, NDPK-B knockdown in cultured ECs also upregulated cellular Ang-2 content. In accordance, NDPK-B depleted ECs display increased protein O-GlcNAcylation similar to diabetic retinae or HG-treated cells. The inhibition of protein O-GlcNAcylation diminished Ang-2 upregulation, confirming that protein O-GlcNAcylation plays a pivotal role in the regulation of Ang-2 levels and hence the development of retinal vasoregression [22]. In this study, we determined whether and how NDPK-B deficiency, similar to HG, alters glucose metabolism towards HBP.

## 2. Materials and Methods

### 2.1. Materials and Reagents

Primary antibodies used were mouse-anti-NDPK-B (MC-412, Kamiya, Seattle, WA, USA, 1:1000), rabbit-anti-NDPK-A (sc-343, Santa Cruz, Heidelberg, Germany, 1:500; detects both NDPK-A and NDPK-B in mouse retinae), mouse-anti-Ang-2 (sc-74403, Santa Cruz, Heidelberg, Germany, 1:500), mouse-anti-O-GlcNAc (ab-2739, Abcam, Cambridge, UK, 1:1000), rabbit-anti-OGA (SAB4200267, Sigma, Munich, Germany, 1:1000), rabbit-anti-OGT (O-6264, Sigma, Munich, Germany, 1:1000), rabbit-anti-N1-phosphohistidine (MABS1330, Millipore, Darmstadt, Germany, 1:1000), mouse-anti-γ-tubulin (T6557, Sigma-Aldrich, Munich, Germany, 1:2000), rabbit-anti-GFAT (obtained in cooperation with Weigert, Tübingen), and sheep-anti-pGFAT (MRC-PPU s343c) for immunoblotting. The secondary antibodies used were rabbit anti-mouse peroxidase (A9044, Sigma-Aldrich, Munich, Germany, 1:20,000), goat-anti-rabbit peroxidase (A9169, Sigma-Aldrich, Munich, Germany, 1:40,000), and donkey-anti-sheep peroxidase (Sigma-Aldrich). qPCR primers were obtained from Applied Biosystems, ThermoFischer: GFAT Hs00899865_m1, OGA Hs00201970_m1, OGT Hs00269228_m1, and 18S Hs03003631_g1. Gelatin from porcine skin (48720, Fluka, Bucharest, Romania) was used as a 1% solution in PBS. Thiamet G (TMG; SML0244, Sigma-Aldrich, Germany) treatment was given at 10 µM for 24 h.

### 2.2. Animals

The use of mice in this study was approved by the local ethics committee (Medical Faculty Mannheim, Heidelberg University, Germany). The care and experimental use of animals were in accordance with institutional guidelines and in compliance with the Association for Research in Vision and Ophthalmology (ARVO) statement. Previous publications describe the generation of NDPK-B^−/−^ mice [23]. Five-month-old male mice were used for the isolation of retinae for the analysis of nucleotide metabolism by Ultra-performance liquid chromatography with Photodiode array detection (UPLC-PDA) and NDPK and OGA enzyme activity assays.

### 2.3. Cell Culture

The use of human umbilical vein endothelial cells (HUVECs) was approved by the local medical ethics committee (Medical Faculty Mannheim, Heidelberg University, Germany). HUVECs were obtained from umbilical cords of healthy newborns with the informed consent of their mothers. The isolation and culture of HUVECs have been previously illustrated [24]. HUVECs were cultured in Endothelial Cell Growth Media (ECGM) supplemented with growth factors (ECGM C-22010, PromoCell, Germany) and 10% Fetal Calf Serum (FCS) (Sigma-Aldrich, Germany) on 1% gelatin-coated flasks. All experiments were performed using cells from passage 1 to 3. HUVECs were used in the study unless otherwise stated.

Murine brain microvascular endothelial cells (MBMECs) were isolated from 10–15-week-old male mice. Three to four brains from the same litter were used for each isolation. MBMECs were isolated as described previously [20]. Isolated MBMECs were suspended in ECGM (ECGM Microvascular (MV) C-22020, Promocell, Germany) supplemented with 10% FCS and 1% antibiotics (P4333, Sigma-Aldrich, Germany). The cells were seeded on a collagen-coated plate and maintained at 37 °C in a humidified incubator. On the following day, endothelial cell selection was performed by incubating the cells for 3 days post-isolation in ECGM + 10% FCS supplemented with 4 µg/mL puromycin (P7255, Sigma-Aldrich, Germany). Cells were characterized by immunofluorescence and used at passage 0 or 1.

### 2.4. siRNA-Mediated Gene Silencing

HUVECs were seeded on gelatin-coated dishes and allowed to attach overnight at 37 °C. The transfection was conducted with the siRNA of choice and lipofectamine RNAiMAX (Life Technologies) in OptiPRO SFM (Thermo Fischer Scientific). Four hours post-transfection (hpt), the transfection media was replaced with ECGM + 10% FCS. The transfected HUVECs were used at 72 hpt. NDPK-B-specific siRNA: AAC UGG UUG ACU ACA AGU CUU (MWG-Eurofins); scramble siRNA: AGG UAG UGU AAU CGC CUU G (MWG-Eurofins); OGA siRNA (SI04159736, Qiagen, Germany); Qiagen scramble (SI03650318, Qiagen, Germany).

### 2.5. Determination of Nucleotide Content via UPLC-PDA

HUVECs were seeded and cultured in 15-cm dishes and subjected to NDPK-B knockdown via siRNA. After 24 hpt, the cells were starved in ECGM + 0.5% FCS for 48 h, then washed with 150 mM ammonium acetate. The dishes were flash-frozen in liquid nitrogen and stored at −80 °C for further metabolic analysis. The retinae from 5-month-old mice were swiftly isolated after sacrificing, flash-frozen in liquid nitrogen and stored at −80 °C until analysis. The determination of nucleotides was adapted from Kochanowski et al. [25]. Either HUVEC cell pellets (10 × 10^6^ cells per sample) or one mouse retina were lysed in 0.37 mL ice-cold 0.5 M perchloric acid with sonication on ice. Samples were neutralized with 86 µL ice-cold neutralization solution (2.5 M KOH in 1.5 M K_2_HPO_4_) after 5 min. sonication. To remove precipitated potassium perchlorate, neutralized samples were centrifuged at 16,400× *g* for 10 min. at 4 °C and filtered with 0.2 µm filters. Nucleotides were immediately analyzed and separated via ion-pair reverse phased chromatography using an Acquity HSS T3 column (150 mm × 2.1 mm, 1.7 µm, Waters) connected to an Acquity H-class UPLC system. The column temperature was set to 40 °C and the column was equilibrated with solvent A (50 mM potassium phosphate buffer, 8 mM tetrabutylammonium hydrogen sulfate, pH 6.5) at a flow rate of 0.45 mL/min. The autosampler temperature was maintained at 6 °C. The elution gradient was as follows: after 2.6 min 0% B (30% acetonitrile in 70% solvent A) to 17 min 77% B, hold for 1 min at 77% B, followed by a return to 0% B and conditioning of the column to initial conditions for 10 min. Nucleotides were detected by an Acquity Photodiode Array (PDA) detector (Waters, 260 nm) and quantified using ultrapure standards (Sigma). Data acquisition and processing were performed with the Empower3 software suite (Waters).

### 2.6. Measurement of Glycolysis and Mitochondrial Respiration 

The measurement of glycolysis and mitochondrial respiration was performed using kits in a Seahorse XFe 96 Analyzer (Glycolysis Stress Test, 103344-100; Mito Stress Test, 103015-100, Agilent Technologies, Germany). Briefly, HUVECs were detached and seeded into pre-coated 96-well plates and allowed to adhere for 2 h at 37 °C. The cells were then washed and starved in non-buffered serum-free Seahorse medium (103334-100, Agilent Technologies, Germany) in a CO_2_-free incubator for 1 h. Glycolysis was monitored as extracellular acidification rate (ECAR), where the data obtained was used to assess glycolysis, glycolytic capacity, or reserve, compared to the controls. Additionally, mitochondrial respiration was monitored as oxygen consumption rate (OCR), assessing basal respiration, respiratory capacity, proton leak, and mitochondrial ATP production. The experiments were conducted and the measurements were obtained according to the standard instructions. The results were analyzed as described [26] using the Wave software and the Seahorse Report Generator (Agilent Technologies, Germany).

### 2.7. Protein Extraction and Immunoblotting

Retinal proteins were extracted in radioimmunoprecipitation assay (RIPA) buffer. Each retina was thoroughly homogenized by passing the suspension through syringe needles of descending diameters (22 G, 25 G, and 27 G). The homogenate was then centrifuged, and the supernatant collected. HUVECs and MBMECs were also lysed in RIPA buffer. Lysates were either used for NDP kinase activity assay, succinylated-wheat germ agglutin (s-WGA) pulldown, or in the assessment of protein expression via immunoblotting. Immunoblotting was performed as previously described [22]. Protein expression was quantified using Image J (NIH, USA).

### 2.8. OGA Activity Assay

OGA activity was measured with a commercially available O-GlcNAcase assay kit (E-130, BMR Service, Utica, NY, USA). Five retinae per genotype or control and NDPK-B-deficient HUVECs were homogenized and the clarified lysates used for the assay. Lysates (10 µL) were subjected to the measurement of OGA activity according to the manufacturer’s instructions.

### 2.9. Quantitative PCR

RNA was isolated from HUVECs homogenized in 1 mL Trizol reagent (Invitrogen, Karlsruhe, Germany) according to the manufacturer’s instructions. RNA (1 µg) was converted to cDNA with Superscript VILO cDNA synthesis kit (11754050, Thermo Fischer, Germany) and subjected to PCR using TaqMan Fast advanced master mix (4444556, Thermo Fischer). The expression of genes was analyzed by the 2(−Delta Delta *C*(T)) (2^−ΔΔ*C*T^) method using MGB-VIC-conjugated 18S:Hs03003631_g1 as a housekeeping control.

### 2.10. s-WGA Pulldown Assay

Succinylated WGA-conjugated agarose beads (SI04159736, Vector Laboratories, UK) were implemented to precipitate O-GlcNAcylated proteins according to the manufacturer’s instructions as previously described [22]. The WGA lysis buffer (50 mM Tris-HCl pH 7.4, 100 mM NaCl, 1% TritonX-100) used contains protease inhibitor (1 tablet/10 mL, Roche). The beads were finally suspended in 1× Laemmli buffer and denatured for 5 min. at 95 °C to extract the immunocomplexes for analysis by immunoblotting.

### 2.11. Adenovirus-Mediated Re-Expression of NDPK-B

HUVECs were transfected with NDPK-B siRNA, starved in ECGM + 0.5% FCS for 24 h, and further infected with recombinant adenovirus encoding green fluorescent protein (Ad-GFP), overexpression of wild type NDPK-B (Ad-NDPK-B-GFP), or overexpression of NDPK-B kinase-dead mutant (Ad-H118N-GFP) in ECGM + 0.5% FCS for 24 h. Cells were monitored for GFP expression for successful and identical transfection in groups and then harvested in RIPA buffer for analysis of protein expression via immunoblotting.

### 2.12. NDP Kinase Activity Assay

HUVECs or retinae were lysed in RIPA buffer and immediately subjected to the NDP kinase activity assay. The test utilizes the transphosphorylase activity of NDPK to generate ATP from GTP and ADP. The newly-formed ATP is directly used by firefly luciferase to convert D-luciferin to the light-emitting oxyluciferin with the Kinase-GLo kit (V6711, Promega, Germany). Under the experimental conditions, the luminescent signal is proportional to the amount of ATP produced, and thus to the enzymatic activity of NDPK. A standard curve was generated by adding increasing concentrations of ATP (0.5–10 µM) in the absence of NDPKs. The NDP kinase activity in cell lysates was measured in a 384-well plate. Lysates were diluted in assay buffer consisting of 50 mM Tris-HCl, pH 7.5, 2 mM MgCl_2_, 1 mM dithiothreitol (DTT), and 0.01% bovine serum albumin (BSA). The readout was commenced by adding the luciferase substrate (V6711, Kinase-GLo, Promega, Germany). After 5 min of incubation, the luminescence was recorded at room temperature using a multiplate reader (EnVision, PerkinElmer, Germany).

### 2.13. Statistical Analysis

Data are presented as mean ± standard deviation (SD). Statistical analyses were performed with the GraphPad Prism 6 software (GraphPad Software, La Jolla). Paired/unpaired student *t*-test or one-way ANOVA with Tukey’s multiple comparison test was used. *p* values < 0.05 were considered to be statistically significant.

## 3. Results

### 3.1. Loss of NDPK-B in ECs Causes an Activation of the HBP

**Figure 1:**
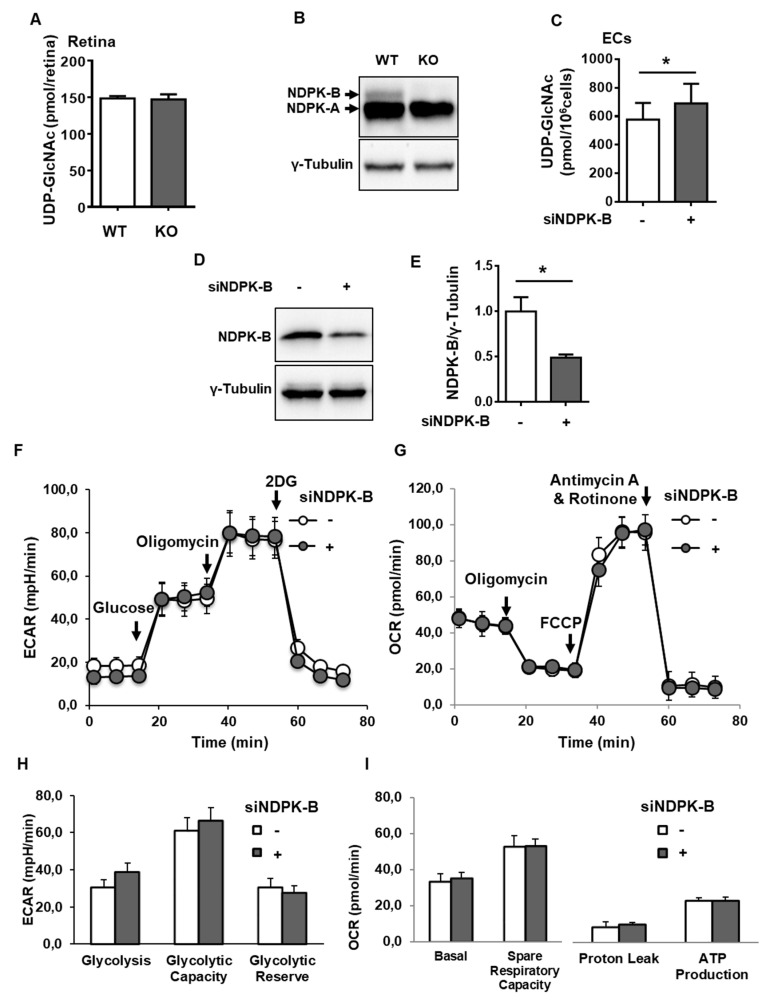
Loss of nucleoside diphosphate kinase B (NDPK-B) in endothelial cells (ECs) causes an activation of the hexosamine biosynthesis pathway (HBP). UDP-GlcNAc was quantified by UPLC-PDA in (**A**) WT and KO retinae from 5-month-old male mice (n = 5 mice per genotype) and (**C**) HUVECs transfected with either scrambled control (−) or siRNA against NDPK-B (siNDPK-B, +) (n = 3). (**B**,**D**) NDPK-B expression in retinae (antibody sc-343) and ECs (antibody MC-412), respectively, was assessed by immunoblot. In NDPK-B-depleted (+) and control HUVECs (−), glycolytic rate and mitochondrial respiration were measured with a Seahorse XF analyzer. (**E**) Quantification of knockdown efficiency between control siRNA and siNDPK-B (n = 3). (**F**) Lactate production from the glycolysis stress test was measured as ECAR. (**G**) Mitochondrial respiration from the Mito stress test was measured as OCR. (**H**) Quantitative comparison of basal glycolysis, glycolytic capacity, and glycolytic reserve (n = 7), and (**I**) quantitative comparison of basal respiration, spare respiratory capacity, proton leak, and ATP production (n = 7). UDP-GlcNAc: uridine diphosphate-*N*-acetylglucosamine; WT: wild type; KO: NDPK-B knockout; ECAR: extracellular acidification rate; OCR: oxygen consumption rate. Results are displayed as mean ± standard deviation (SD). *: *p* < 0.05.

To study a possible regulation of the HBP by NDPK-B, we first examined the HBP end product, UDP-GlcNAc, in NDPK-B-deficient retinae as well as in NDPK-B-depleted ECs by UPLC-PDA. Our previous studies revealed an elevation of Ang-2 in primary human retinal microvascular ECs and HUVECs by HG and NDPK-B depletion [21,22,27]. HUVECs were applied in this study due to their accessibility. Successful knockout and knockdown of NDPK-B in the retina and ECs, respectively, was confirmed by immunoblotting (Figure 1B,D). Approximately 150 pmol of UDP-GlcNAc was detected per retina. This UDP-GlcNAc level was not altered in KO retinae as compared to the wild type retinae (Figure 1A). In cultured ECs, about 580 pmol UDP-GlcNAc was detected per 10^6^ cells. NDPK-B knockdown significantly increased UDP-GlcNAc levels by about 20% (Figure 1C). Since the retina is a complex organ consisting of many cell types, of which ECs are a small subset, the data indicate that NDPK-B deficiency might activate the HBP in a cell type-specific manner. Increased flux through glycolysis is known to also elevate HBP flux [7,28]. As such, we measured glycolysis and mitochondrial respiration in NDPK-B depleted ECs. Glycolysis was monitored as extracellular acidification rate (ECAR). NDPK-B-depleted ECs acted similarly to control cells and did not exhibit altered glycolysis, glycolytic capacity, or reserve (Figure 1F,H). Mitochondrial respiration was monitored as oxygen consumption rate (OCR). Control ECs showed basal respiration at around 50 pmol/min (Figure 1G). NDPK-B depletion did not alter basal respiration, respiratory capacity, proton leak, or mitochondrial ATP production as compared to the controls (Figure 1G,I). Collectively, these data show that NDPK-B depletion in ECs did not alter glycolysis and mitochondrial respiration, but resulted in an elevation of UDP-GlcNAc. This indicates an activation of the HBP in NDPK-B depleted ECs.

### 3.2. Loss of NDPK-B in ECs Alters Nucleotide Metabolism without Changing NTP Levels

**Figure 2:**
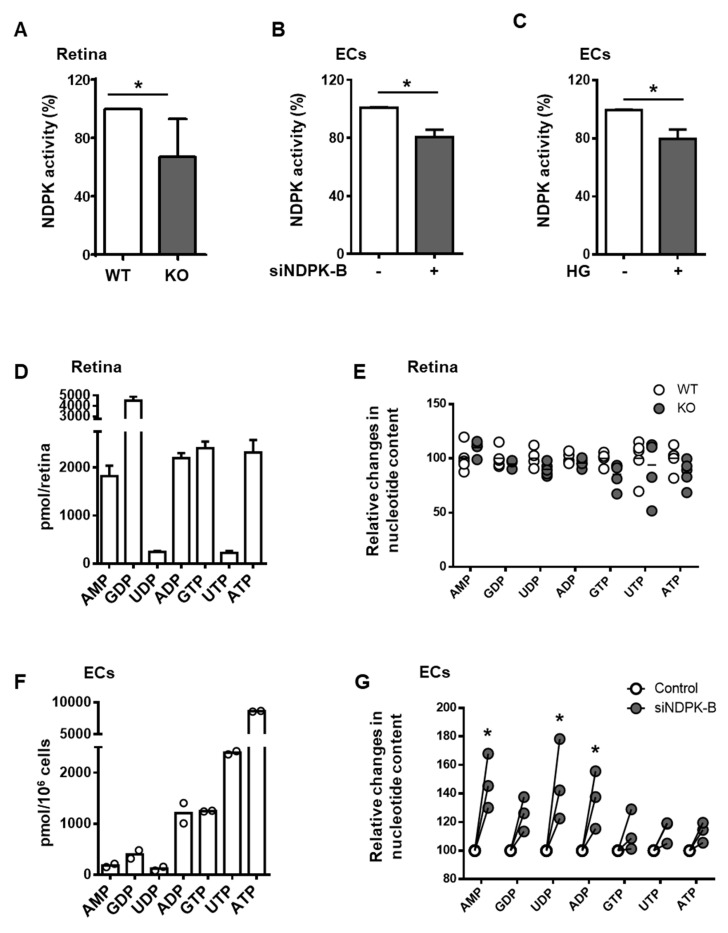
Loss of NDPK-B in ECs alters nucleotide metabolism without changing NTP levels. (**A**) NDPK activity in lysates of WT and KO retinae (n = 10 mice per genotype). (**B**) NDPK activity in HUVECs transfected with either scrambled control (−) or siRNA against NDPK-B (siNDPK-B, +) (n = 8). (**C**) NDPK activity in HUVECs treated with either normal (−) or high glucose (HG, +) (n = 9). (**D**) UPLC-PDA analysis of absolute values of nucleotide composition in the retina in pmol/retina. (**E**) Quantification of the indicated nucleotides between WT and KO retinae by UPLC-PDA (n = 5 mice per genotype). (**F**) UPLC-PDA analysis of absolute values of nucleotide composition to assess the proportional contents of the nucleotides in HUVECs, presented in pmol/10^6^ cells (n = 2). (**G**) Relative comparison of nucleotides in control transfected and NDPK-B depleted HUVECs (n = 3). Mean values in control cells were set to 1.0. WT: wild type; KO: NDPK-B knockout, HUVECs: human umbilical vein endothelial cells. Results are displayed as mean ± SD; *: *p* < 0.05.

Since NDPKs catalyze the formation of NTPs, including UTP, we analyzed the effect of NDPK-B deficiency in the retina and ECs on the overall NDP kinase activity and nucleotide levels. The overall NDP kinase activity was reduced by 35% in the KO retina (Figure 2A) and 20% in the siNDPK-B-treated ECs (Figure 2B). This reduction is similar, interestingly, to the reduction in NDPK activity seen upon HG stimulation in ECs (Figure 2C).

To determine whether NDPK-B depletion activates HBP via altered nucleotide metabolism, we determined nucleotide levels via UPLC-PDA in NDPK-B deficient retinae and ECs. In the retina, the highest nucleotide levels were observed for GDP at 4500 pmol/retina and GTP at 2500 pmol/retina (Figure 2D). This likely reflects the important role of guanine nucleotides in signal transduction in the photoreceptors of the retina [29]. Retinae contain rather low amounts of UTP/UDP compared to GTP/GDP. No differences in nucleotide content between KO and WT retinae were detected (Figure 2E). In contrast, cultured ECs exhibit a completely different nucleotide content profile than the total retina. They are higher in NTPs (in particular the general energy donor, ATP) at approximately 7500 pmol/10^6^ cells, followed by UTP and GTP at 1500 and 1800 pmol/10^6^ cells, respectively (Figure 2F). NDPK-B knockdown ECs did not show differences in NTP levels but displayed a relative increase in AMP, UDP, and ADP levels by about 50% (Figure 2G). This accumulation of NDPs indicates a slower nucleotide metabolism due to the loss of one of the major NDPK isoforms. Nevertheless, as the NTP pool was unchanged, the data also imply that NDPK-B depletion in ECs may be compensated for by other isoforms, at least for the upkeep of NTP formation from ATP and the respective NDP. This includes UTP as an important co-factor for the formation of UDP-GlcNAc. Loss of NDPK-B in ECs causes an activation of the HBP.

### 3.3. Loss of NDPK-B in ECs Increases GFAT Content and Activity

**Figure 3:**
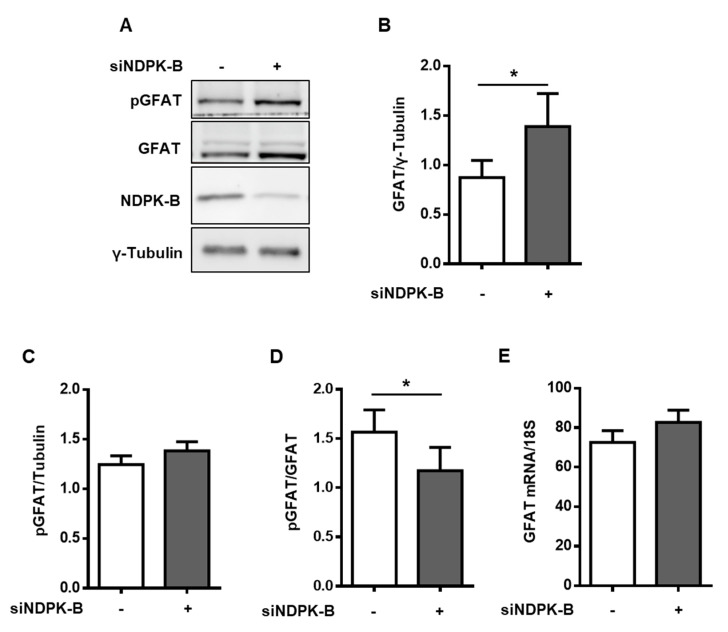
Loss of NDPK-B in ECs increases GFAT content and activity. (**A**) HUVECs transfected with either scrambled control (−) or siRNA against NDPK-B (siNDPK-B, +) were analyzed for NDPK-B, GFAT, and GFAT phosphorylated at Ser243 (pGFAT) content by immunoblotting. (**B**) Quantification of the GFAT content relative to loading control, γ-tubulin. (**C**) Quantification of pGFAT content relative to loading control, γ-tubulin. (**D**) Quantification of pGFAT/GFAT. (**E**) qPCR quantification of GFAT mRNA expression relative to 18S. GFAT: glutamine:fructose-6-phosphate amidotransferase. Results are presented as mean ± SD from n = 5 independent experiments. *: *p* < 0.05.

GFAT is a rate-limiting enzyme in the HBP, catalyzing the conversion of fructose-6-phosphate to glucosamine-6-phosphate, an important precursor of UDP-GlcNAc. To investigate the role of GFAT, we first examined its protein content in NDPK-B knockdown ECs (Figure 3A). GFAT was upregulated nearly 1.7-fold in NDPK-B-depleted ECs as compared to the controls (Figure 3A,B). It has recently been reported that the level of phosphorylation of endothelial GFAT at serine 243 is inversely correlated with its enzymatic activity and results in a corresponding alteration of subsequent protein O-GlcNAcylation [30,31]. In the NDPK-B-depleted cells, GFAT phosphorylation at serine 243 was significantly reduced by about 35% as compared to the controls, indicating an enhancement of GFAT activity (Figure 3B,D), although the absolute phosphorylation of GFAT remained unaltered (Figure 3C). However, the amount of mRNA encoding GFAT, measured via qPCR, remained unaltered (Figure 3E), pointing to a post-transcriptional mechanism causing the elevation of GFAT levels upon NDPK-B knockdown. Taken together, the data indicate that the upregulation of GFAT content and activity likely account for the increase in the UDP-GlcNAc level upon NDPK-B depletion as shown above.

### 3.4. Reduced OGA Activity Enhances Protein O-GlcNAcylation in NDPK-B Depleted ECs 

**Figure 4:**
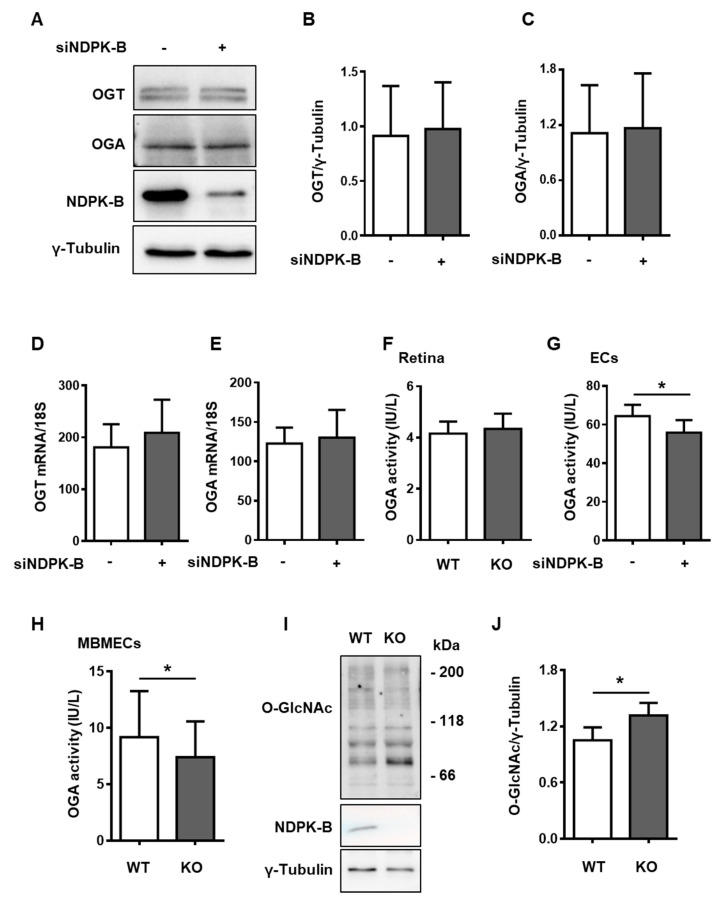
The reduction of OGA activity contributes to enhanced protein O-GlcNAcylation in NDPK-B-depleted ECs. (**A**) HUVECs transfected with either scrambled control (−) or siRNA against NDPK-B (siNDPK-B, +) were analyzed for OGT, OGA, and NDPK-B content by immunoblotting (n = 9). (**B**) Quantification of OGT and (**C**) OGA relative to loading control, γ-tubulin. (**D**) mRNA levels of OGT and (**E**) OGA relative to 18S quantified by qPCR (n = 6). OGA activity in (**F**) WT and KO retinae isolated from 5-month-old male mice (n = 8 mice per genotype), (**G**) lysates of control and NDPK-B knockdown HUVECs (n = 7) and (**H**) lysates of WT and KO MBMECs isolated from 10–15-week-old WT and KO mice. (**I**) NDPK-B and protein O-GlcNAc content analyzed by immunoblot in the same lysates of WT and KO MBMECs. (**J**) Quantification of protein O-GlcNAcylation relative to γ-tubulin (n = 5). MBMECs were prepared from 3–4 animals per genotype. WT: wild type; KO: NDPK-B knockout; OGT: O-GlcNAc transferase; OGA: O-GlcNAcase; MBMECs: murine brain microvascular ECs. Results are presented as mean ± SD. *: *p* < 0.05.

UDP-GlcNAc is the substrate for OGT in the protein O-GlcNAcylation cycle, which is controlled by the enzymes OGT and OGA. We therefore assessed the content of OGT and OGA in NDPK-B depleted ECs (Figure 4A). OGT and OGA protein levels were unaltered between knockdown and control ECs (Figure 4B,C). Similarly, qPCR data showed that the content of mRNA encoding OGT or OGA was not changed in NDPK-B depleted ECs as compared to the control cells (Figure 4D,E).

As the functional activity of OGA is not only regulated by its expression level, we further assessed the enzyme activity of OGA in KO retinae and NDPK-B knockdown ECs. No obvious change in OGA activity was detected between the NDPK-B KO and WT retinae (Figure 4F). In ECs, NDPK-B knockdown caused a significant 12% reduction in OGA activity as compared to the controls (Figure 4G). In order to investigate whether NDPK-B depletion-induced alteration in OGA activity and protein O-GlcNAcylation is a general phenomenon in ECs, particularly in microvascular ECs, murine brain ECs (MBMECs) were isolated from NDPK-B KO mice and assessed [20]. Immunoblotting experiments verified the absence of NDPK-B in MBMECs isolated from the KO mouse line (Figure 4I). KO MBMECs exhibited significantly lower (approximately 12.5% lower) OGA activity than WT MBMECs (Figure 4H). Similar to NDPK-B depletion in ECs, protein O-GlcNAc was significantly higher in KO than in WT MBMECs (Figure 4I,J). These data reveal that the increased formation of UDP-GlcNAc via the HBP and a reduction of OGA activity likely act synergistically in the elevation of protein O-GlcNAcylation upon the loss of NDPK-B in ECs.

### 3.5. Enhanced Protein O-GlcNAcylation Increases Ang-2 Content 

**Figure 5:**
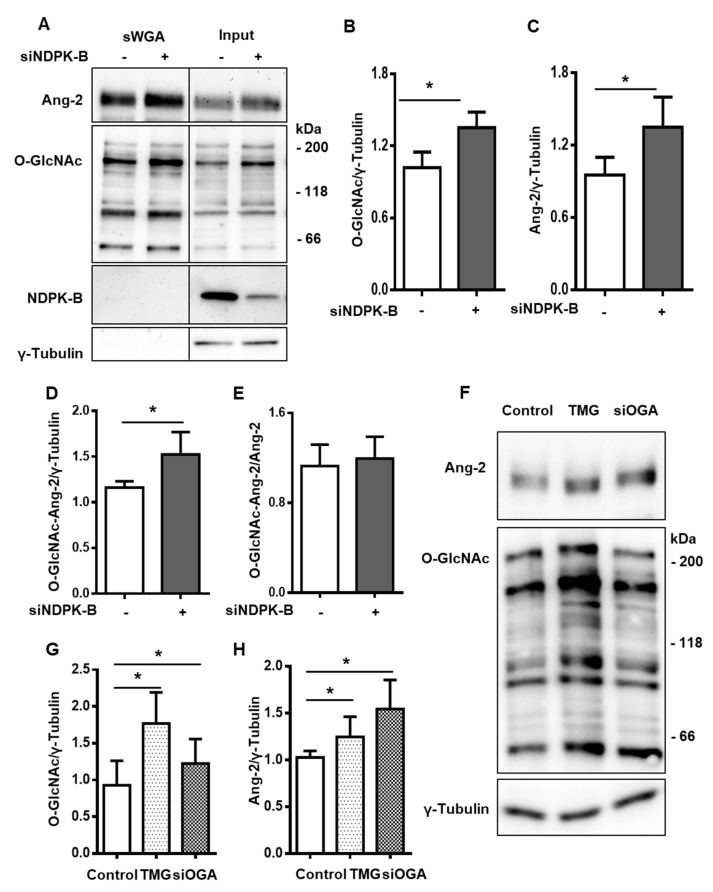
Elevation of O-GlcNAcylation by the suppression of OGA activity regulates Ang-2 expression. (**A**) HUVECs were transfected with either scrambled control (−) or siRNA against NDPK-B (siNDPK-B, +) and O-GlcNAcylated proteins were precipitated from the lysates using s-WGA beads. Precipitates (s-WGA) and whole cell lysates (Input) were subjected to immunoblotting and probed for Ang-2, O-GlcNAc, NDPK-B, and γ-Tubulin (n = 4). Quantification of (**B**) protein O-GlcNAcylation, (**C**) Ang-2, and (**D**) O-GlcNAcylated Ang-2 in the precipitates relative to γ-tubulin in the input lysates. (**E**) Quantification of O-GlcNAcylated Ang-2 relative to Ang-2 in the input lysates. (**F**) HUVECs were transfected with either scrambled control or siRNA against OGA (siOGA), or treated with 10 µM of the OGA inhibitor, TMG, for 24 h. Cell lysates were analyzed for Ang-2 and protein O-GlcNAcylation by immunoblot (n = 8). Quantification of (**G**) protein O-GlcNAcylation and (**H**) Ang-2 relative to γ-tubulin. S-WGA: succinylated WGA-bound agarose beads; TMG: Thiamet G; OGA: O-GlcNAcase. Results are presented as mean ± SD. *: *p* < 0.05.

Prior findings indicate that Ang-2 is a crucial mediator of vasoregression in NDPK-B deficiency and its content is regulated by O-GlcNAcylation [21,22]. In accordance with the published data, total protein O-GlcNAcylation was significantly increased in NDPK-B-depleted ECs as compared to the control cells. O-GlcNAcylated proteins were enriched using s-WGA beads, which specifically recognize the O-GlcNAc moiety, and the overall increase in protein O-GlcNAcylation was reflected in the bound fraction (Figure 5A,B). NDPK-B was not O-GlcNAcylated in ECs. In line with the reported data [21,22], the Ang-2 content was significantly higher in NDPK-B-depleted ECs as compared to control ECs (Figure 5A,C). Notably, Ang-2 turned out to be O-GlcNAcylated under basal conditions, since Ang-2 was detectable using specific antibodies in the s-WGA immunoprecipitated proteins. We also attempted immunoprecipitation using Ang-2 antibodies, but were unsuccessful due to their unspecific nature. The amount of O-GlcNAcylated Ang-2 was nearly 1.5-fold higher in NDPK-B-depleted ECs than in the respective controls (Figure 5A,D). With respect to the total Ang-2 level in the cell lysate, the relative fraction of O-GlcNAcylated Ang-2 was unchanged between NDPK-B-depleted and control cells (Figure 5E). To determine if the upregulation of Ang-2 in NDPK-B-depleted ECs is the result of the dysregulation of OGA, we modulated OGA with the potent OGA-specific inhibitor Thiamet G (TMG). TMG treatment significantly increased protein O-GlcNAcylation by two-fold (Figure 5F,G), and Ang-2 content was significantly upregulated in TMG by 1.3-fold (Figure 5F,H). To confirm the data on the regulation of Ang-2 by cellular O-GlcNAcylation, we concomitantly performed siRNA-mediated OGA knockdown experiments. Similar to TMG treatment, protein O-GlcNAcylation in the OGA knockdown cells was 1.25-fold higher as compared to the control siRNA treated cells (Figure 5F,G). Ang-2 content was significantly upregulated in siOGA treated cells by 1.5-fold, more effectively than through TMG treatment (Figure 5H). The data indicate that Ang-2 levels in ECs are susceptible to changes in cellular O-GlcNAcylation.

### 3.6. Regulation of Protein O-GlcNAcylation Is Independent of the Enzymatic NTP/NDP Transphosphorylase and Protein Histidine Activity of NDPK-B Kinase

**Figure 6:**
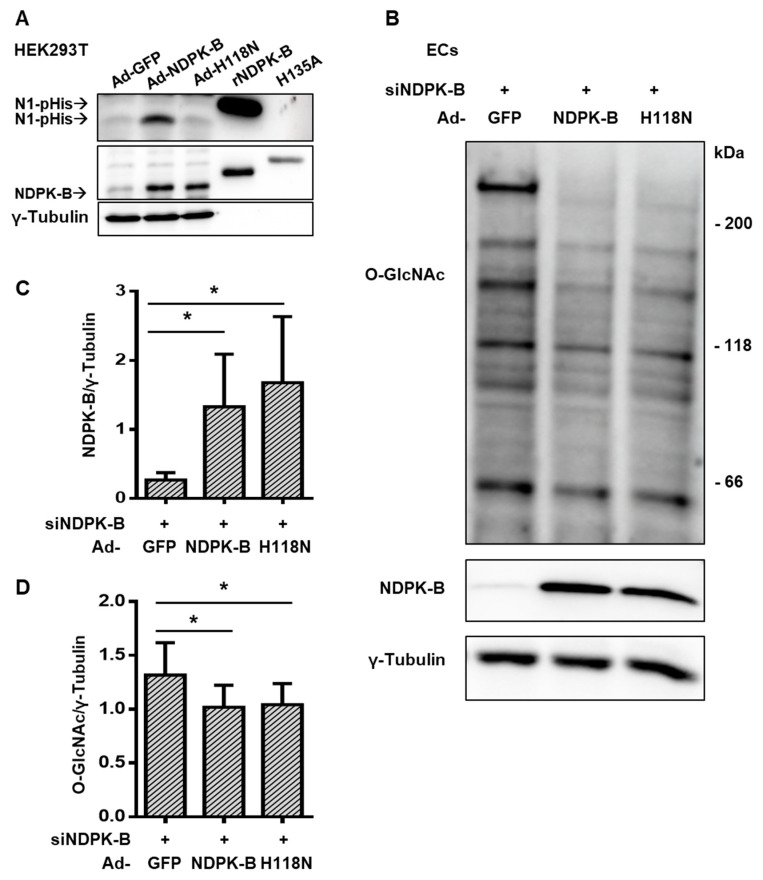
Regulation of protein O-GlcNAcylation is independent of the enzymatic NTP/NDP transphosphorylase and protein histidine activity of NDPK-B kinase. (**A**) HEK293T cells were transduced with adenoviruses expressing GFP (Ad-GFP), NDPK-B (Ad-NDPK-B), or enzymatically inactive mutant NDPK-B H118N (Ad-H118N). Cell lysates were analyzed for N1-phosphohistidine, NDPK-B, and γ-tubulin content by immunoblot. Recombinant NDPK-B (rNDPK-B with 6× His-Tag) and a recombinant, enzymatically inactive mutant NDPK-C (H135A with 6× His-Tag) were used as positive and negative controls, respectively, for NDPK autophosphorylation. (**B**) HUVECs were transfected with siRNA against NDPK-B (siNDPK-B, +) and then infected with adenoviruses expressing either GFP (Ad-GFP), NDPK-B (Ad-NDPK-B), or NDPK-B H118N (Ad-H118N). NDPK-B and protein O-GlcNAcylation were analyzed by immunoblot (n = 8). Quantification of (**C**) NDPK-B and (**D**) protein O-GlcNAcylation relative to γ-tubulin. Results are presented as mean ± SD. *: *p* < 0.05.

Besides its NTP/NDP transphosphorylase [17] and its protein histidine kinase activity [32], which are both dependent on the presence of a histidine residue in position 118 (H118) of NDPK-B, NDPK-B can also function as a scaffold protein, regulating, for example, caveolae formation in ECs [19,20]. To determine whether the enzymatic activities of NDPK-B are required for the regulation of protein O-GlcNAcylation in ECs, we performed experiments with adenoviral re-expression of either wild type or an enzymatic inactive mutant H118N in NDPK-B-depleted ECs. We used an N1 phosphohistidine-specific antibody (N1-pHis) to monitor the catalytic activity of NDPK-B [32]. To validate the adenoviruses used, we first transduced HEK293T cells. Similar levels of NDPK-B overexpression were achieved with Ad-NDPK-B and Ad-H118N (Figure 6A). Purified recombinant NDPK-B (rNDPK-B) and a recombinant kinase activity-deficient mutant of NDPK-C (H135A) served as a positive control and as a negative control, respectively. In cells transfected with a control virus (Ad-GFP), the detection of N1-pHis correlated to the modest expression of endogenous NDPK-B. A strong increase in NDPK-B autophosphorylation occurred only in Ad-NDPK-B, but not in Ad-H118N transduced cells. Therefore, the constructs were used to re-express NDPK-B in siRNA-mediated knockdown ECs. A similar overexpression of NDPK-B, by more than five-fold, was achieved in Ad-NDPK-B and Ad-H118N infected cells (Figure 6B,C). Both re-expression of wild type NDPK-B or its kinase-inactive mutant diminished overall protein O-GlcNAcylation to a similar extent (by about 23%) from the NDPK-B-depleted ECs (Figure 6B,D). Collectively, the data show that the regulation of protein O-GlcNAcylation by NDPK-B is independent of its NTP/NDP transphosphorylase and protein histidine phosphorylation activity.

## 4. Discussion

In this study, the relationship between NDPK-B and protein O-GlcNAcylation in ECs was investigated. The data showed that the depletion of NDPK-B in ECs: (1) is involved in the activation of the HBP without altering glycolysis; (2) does not alter nucleotide triphosphate levels, despite altering nucleotide metabolism; (3) increases the level of the rate-limiting enzyme GFAT in the HBP in ECs and diminishes its inhibitory phosphorylation at Ser243, promoting UDP-GlcNAc production; (4) is further associated with a reduction of OGA activity; and (5) enhances NDP kinase-independent protein O-GlcNAcylation that drives the upregulation of Ang-2, which itself is O-GlcNAcylated.

These data indicate the importance of the HBP in vascular damage related to NDPK-B-deficient ECs [21,22] and reveals further mechanisms behind this established vasoregressive model, lending it further credibility and validation for mechanistic investigations related to vasoregressive retinopathy. It has been shown before that high glucose (HG) leads to the activation of the HBP and an increase in UDP-GlcNAc levels in ECs [7,28]. As a result of the HBP activation, protein O-GlcNAcylation is increased in diabetic retinae or HG-treated ECs [7,16,21,22]. As also illustrated herein, by interfering with OGA activity, the level of protein O-GlcNAcylation governs Ang-2 content in NDPK-B depleted ECs and in the NDPK-B deficient retinae [21,22]. Additionally, in MBMECs isolated from NDPK-B-deficient mice, enhanced protein O-GlcNAcylation was clearly evident. The upregulation of Ang-2 upon NDPK-B depletion is seemingly common to ECs of all vascular beds; as illustrated in these studies, it also occurs in human microvascular ECs from the dermis and in the retina, in addition to HUVECs [21,27]. The increase in Ang-2 content and release has been shown to be responsible for the initiation of pericyte dropout in the retina and the formation of acellular capillaries, and thus vasoregression [13,14,15], a process recently also confirmed to occur in NDPK-B-deficient mice [21,22]. Several possibilities as to how protein O-GlcNAcylation increases Ang-2 levels have been suggested. For instance, as mentioned previously, hyperglycemia promotes the methylglyoxal modification of mSin3A in ECs, triggering increased O-GlcNAcylation of the transcriptional regulator Sp3 via recruitment of OGT [12]. We and others showed that Ang-2 expression in ECs is dependent on FoxO1, a transcription factor that is also stabilized by O-GlcNAcylation [22,33]. This study demonstrates that Ang2 expression is controlled by cellular O-GlcNAcylation. The differences in regulation extent of Ang-2 by OGA inhibitor TMG and OGA siRNA might be due to an unknown off-target effect of TMG or the multiple regulation potential of Ang-2. Another feature, which became apparent in this study, is that Ang-2 itself is highly O-GlcNAcylated. O-GlcNAcylation of a protein is often associated with increased stability of the protein due to the inhibition of proteasomal degradation [11,34]. However, O-GlcNAcylated Ang-2 was not changed between NDPK-B-depleted and control ECs. Therefore, a rather complex regulation of Ang-2 levels in ECs can be envisioned. Taking into account that the upregulation of GFAT, as well as the suppression of the OGA activity, were independent of gene transcription, many of the alterations similarly induced by hyperglycemia/high glucose and loss of NDPK-B are caused by changes in post-transcriptional modification patterns. Thus, the involvement of NDPK-B-mediated regulation of translational machinery is not excluded.

The increase in protein O-GlcNAcylation, as well as the upregulation of Ang-2 caused by NDPK-B deficiency, has been detected previously in whole retinal lysates [21]. The increase in UDP-GlcNAc levels, which was identified upon NDPK-B depletion in cultured ECs, was however not detected in NDPK-B-deficient retinae. The retina is a complex organ consisting of many cell types, of which ECs are a small subset. Therefore, changes in a small subset of cells may escape detection, especially when the detection method used analyses components which are present in most cell types, such as nucleotides or their derivatives. Hypothesizing that the elevation of UDP-GlcNAc upon NDPK-B deficiency is specific to ECs and maybe the Müller glia, another source of protein O-GlcNAcylation-induced Ang-2 in the retina [35] could explain how it escaped detection in the pool of total UDP-GlcNAc in the retinal lysate. This interpretation is corroborated by the results obtained from the nucleotide content analysis. While GTP and GDP were the most prominent nucleotides in the total retinal lysates, ATP showed by far the highest level in ECs, which is consistent with their known high glycolysis rate, utilizing ATP as a source of energy [36]. Moreover, none of the changes in the nucleotide pattern observed in NDPK-B-depleted ECs were reflected in total lysates of NDPK-B deficient retinae.

The NDPKs are originally a family of housekeeping enzymes that replenish the NTP pool via their NTP/NDP transphosphorylase activity [17,18]. As NDPK-B is one of the two major isoforms, its depletion in ECs diminished the overall NDP kinase activity by 20%. Correspondingly, 35% of the NDP kinase activity was missing from the retinae of NDPK-B-deficient mice. Nevertheless, although the absence of NDPK-B slightly altered the nucleotide metabolism in ECs, the measured NTP levels were not changed, indicating that the activity of the remaining NDPK family members is sufficient to stabilize these levels. An altered UTP level [37] therefore has to be excluded as the driving force for the enhanced UDP-GlcNAc levels upon NDPK-B depletion. In line with this interpretation, the re-expression experiments with NDPK-B or its catalytically inactive mutant showed that its enzymatic activity is not required to lower protein O-GlcNAcylation in ECs. The scaffolding functions of NDPK-B in the regulation of protein complex formation have been reported before and are also seen for other NDPK isoforms, e.g., NDPK-C [38,39,40,41]. In ECs, NDPK-B is involved in caveolae formation and caveolin transport [19,20]. Thus, the possible contribution of Cav-1 in NDPK-B-dependent vasoregression needs to be assessed and might help decide whether the overexpression of NDPK-B in wild type and kinase-dead mutant forms is an option to be considered to tackle the Ang-2 upregulation in the retina as a possible therapeutic approach to treat vasoregression.

**Figure 7:**
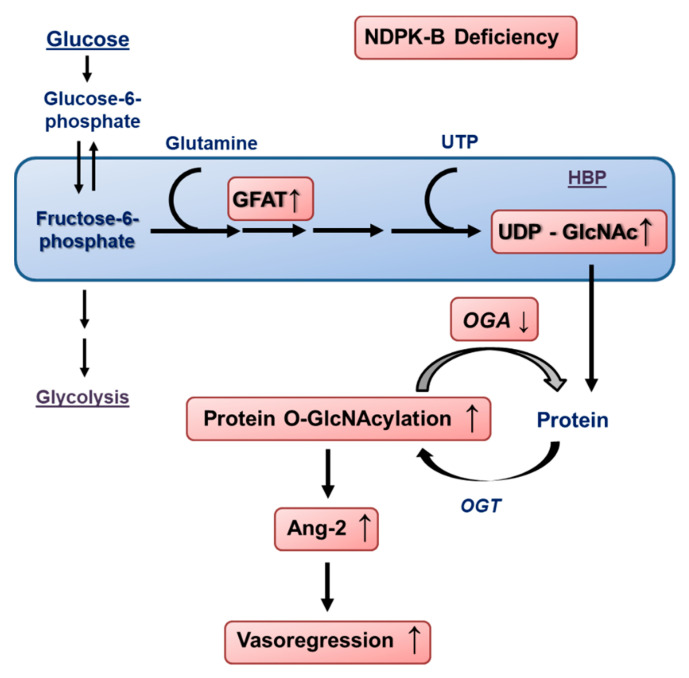
Schematic overview of the activation of the HBP and enhanced protein O-GlcNAcylation caused by the loss of NDPK-B in the endothelium and the subsequent upregulation of Ang-2. Loss of NDPK-B increases the HBP flux via the upregulation of GFAT, leading to an increase in UDP-GlcNAc production. The concomitant reduction in OGA activity results in an accumulation of O-GlcNAcylated proteins, driving the upregulation of Ang-2 in the microvascular endothelium of the retina, thereby inducing vasoregression.

In conclusion, the data reported herein shed light on a novel pathway by which the loss of NDPK-B in ECs causes activation of the HBP. By increasing the GFAT content and suppression of OGA activity, protein O-GlcNAcylation is enhanced, further upregulating Ang-2 (Figure 7). The enhanced secretion of Ang-2 induces pericyte loss, acellular capillary formation, and retinal vasoregression.

## Abbreviations

NDPK-B: nucleoside diphosphate kinase B; Ang-2: angiopoietin-2; HBP: hexosamine biosynthesis pathway; OGA: O-GlcNAcase; O-GlcNAc: O-linked *N*-acetylglucosamine; ECs: endothelial cells.
